# Supplementary material for: A Simulation to Improve Understanding and Communication of Ethical Dilemmas That Surround Brain Death
Source: MedEdPORTAL. 2024 Sep 26;20:11444. doi: 10.15766/mep_2374-8265.11444 (PMC11424717; doi:10.15766/mep_2374-8265.11444)
Supplement: Supplementary file 1 — Prebrief Instructions and Presentation.pptxStandardized Patient Case Development Tool.docxSimulation Case.docxWBUH Checklist for Determining Brain Death.docxInstructions for Debrief.docxQuestionnaire.docx [file mep_2374-8265.11444-s001.zip › D. WBUH Checklist for Determining Brain Death.docx]

**Appendix D. William Beaumont University Hospital Checklist for Determining Brain Death***

- 1. A core body temperature of greater than or equal to 35 degrees Celsius (95 degrees Fahrenheit) must be achieved and maintained during examination and testing to determine death.
  2. Coma characterized by absence of spontaneous or induced cerebral motor response to painful stimulation that is applied within the cranial nerve distribution. Decerebrate and decorticate responses or seizures are absent.
  3. Absence of pupillary response to a bright light is documented in both eyes. Note that the pupils are usually fixed in a mid-size or dilated position (4–9 mm). Constricted pupils suggest the possibility of drug intoxication.
  4. Absence of corneal reflexes.
  5. Absence of spontaneous eye movements.
  6. Absence of eye movements in response to vestibular stimulation by cold caloric testing (50 mL ice water each side; 5-minute interval between sides).
  7. Absence of eye movements in response to oculocephalic reflex testing.
  8. Absence of gag reflex in response to stimulation of posterior pharynx.
  9. Absence of cough reflex in response to deep bronchial suctioning.
  10. Absence of spontaneous respiration at pCO2 greater than or equal to 60 mmHg and an increase in pCO2 of greater than or equal to 20mmHg above pre-apnea test level. Evident respiratory acidosis at the completion of the apnea test.
      1. For adults, the apnea test must be done using the apneic diffusion oxygenation technique to prevent hypoxia performed with a 10cmH2O peep valve. The body temperature must be at 35°C or higher before the apnea test.
      2. The patient is to be monitored during the exam with arterial blood gases determination. The certifying physician must continuously observe the patient for any respiratory effort throughout administration of the test.

*Note: The checklist for determining brain death varies between institutions. The above checklist represents the steps taken for trainees at William Beaumont University Hospital. Educators should use their own institutional checklists for determining brain death.
