# Supplementary material for: A Simulation to Improve Understanding and Communication of Ethical Dilemmas That Surround Brain Death
Source: MedEdPORTAL. 2024 Sep 26;20:11444. doi: 10.15766/mep_2374-8265.11444 (PMC11424717; doi:10.15766/mep_2374-8265.11444)
Supplement: Supplementary file 1 — Prebrief Instructions and Presentation.pptxStandardized Patient Case Development Tool.docxSimulation Case.docxWBUH Checklist for Determining Brain Death.docxInstructions for Debrief.docxQuestionnaire.docx [file mep_2374-8265.11444-s001.zip › E. Instructions for Debrief.docx]

**Appendix E. Instructions for Debrief**

**Defusing**

Because the simulation may feel confrontational given the skeptical and, at times, argumentative tone of the spouse, the defusing step allows trainees to express some of their strong emotions (e.g., frustration, anger, disappointment, or surprise). This provides a space to decompress so that the trainees can absorb the additional content from the debrief. Facilitators should not offer guidance or teaching during this stage. While some of the trainees’ reactions may come in the form of a question (e.g., What should I have done when the spouse asked for the patient to be full code?), it best to leave the didactics and discussion of best practices to the ‘discovery’ portion (see below).

1. Begin by asking trainees to share their feelings about the simulation they just experienced or observed.
2. Encourage the trainees who observed the simulation to give feedback to the trainee who participated in the simulation. In our experience this was often in the form of, “I thought you did _____ really well” and also, “I would have reacted the same way if the spouse said ____.”
3. Ask the confederate to share how they felt during the simulation (e.g., did they think it was confrontational? Did the trainee show empathy?).

**Discovery**

Discovery should proceed through an ask-answer-discuss framework. Start by prompting the trainees to identify, explore, and analyze the mental models or frames that guide their behavior during the brain death examination. Then, discuss and compare their mental models with policy statements and recommendations. Opening space for discussion before the presentation of policy and consensus positions serves to actively engage the trainees. We found the discovery stage was a valuable time for those who had experience with brain death to share their insights with their colleagues. Those who were inexperienced benefitted from talking with their colleagues who were more comfortable talking about brain death and its ethical issues. Discovery should include three topics: clinical/legal, communication, and ethical/philosophical

1. Clinical/legal: Ask the following questions, discuss, and then present hospital policy and consensus positions from the American Academy of Neurology^1^ and the World Brain Death Project.^2^
   1. Is surrogate consent required to perform an examination for brain death?
      1. Note: We observed that trainees were commonly confused on what to do if the spouse refused to consent to the brain death examination. Many felt like consent was required before proceeding and that they were not prepared to appropriately react to a surrogate when they declined further testing.
   2. How should the medical team proceed if the surrogate disputes the brain death determination?
      1. Note: We observed that many trainees were uncomfortable having a discussion about a view of death (i.e., one that relies on the heartbeat and not brain function) that is not recognized within medicine. For many trainees this was their first experience talking about this non-standard view.
   3. Should a ‘full code’ status remain in place after brain death has been declared?
2. Communication: Divide into three contexts: physician introduction, delivery of diagnosis, and surrogate dispute.
   1. *Introduction:* Start with an open question such as, “What do you think would be an effective way of introducing yourself to the spouse?” Then, offer suggestions on how to greet family when the trainee first encounters the family of a comatose patient. For example, saying “I am sorry to meet you in these difficult circumstances. Please excuse me while I conduct my examination. I will discuss my findings with you after this has been completed.”
   2. *Delivery of diagnosis:* Ask trainees to reflect back on the simulation and identify language during the delivery of the diagnosis that could be confusing for family members and/or surrogate decision makers. Emphasize that communication should be succinct and jargon free. Instead of saying that the patient is “brain dead,” the trainee should say that the patient has “died.” Similarly, saying “machines” instead of “life support” avoids the contradiction of providing *life* support to a deceased person. The discussion of language is a good opportunity for the trainees to reflect on their use of colloquial language and how they should be aware of how they may sow confusion among the family.
   3. *Surrogate Dispute:* The dispute portion should start with the open-ended question, “How would you suggest resolving a dispute between doctor and surrogate in this context?” Follow the discussion with a presentation of the different kinds of surrogate objections that typically arise (e.g., informational, emotional, principled) and suggestions for how to respond to each category.^3^ Offer strategies on how to resolve the dispute if it becomes intractable. The trainees should be made aware that there are services that may be at their disposal that can aid them in these types of situations (i.e., legal and ethical services).
3. Ethical/philosophical: This section is more didactic than the clinical/legal and communication portions. Trainees will likely have less experience with the ethical/philosophical elements of brain death, so to help facilitate learning, start by introducing a topic and then follow it with discussion.
   1. Set up the discussion of a proposed conscience clause for death by introducing the three main competing views of death. The whole brain view, which is currently the medico-legal standard, says that death occurs when there has been irreversible cessation of all functions of the brain, including the brain stem. In contrast, the high-brain view says that death occurs when there is irreversible cessation of function of the cerebral hemispheres (i.e., irreversible loss of consciousness). Finally, the circulatory view states that death occurs when there has been irreversible cessation of cardiopulmonary function.
   2. Present the theoretical proposal of a conscience clause for death, which can be summarized in the following: There are reasonable disagreements on the definition of death, i.e., the whole-brain, high-brain, and circulatory views outlined above. Such disagreements are not based on *scientific* reasons, but rather arise from philosophical or religious commitments about the necessary functions of life. For example, a surrogate may agree with all the scientific reasons for believing a patient has irreversibly lost all functions of the brain and brain stem (e.g., physical exam, lab tests, and imaging), and yet believe the patient is still alive because they maintain a necessary function of life (i.e., heart beat). Public policy should respect a pluralism of views when there is reasonable disagreement. Therefore, individuals ought to have a choice between the whole-brain, high-brain, and circulatory view of death.^4^ For a more in-depth discussion of the conscience clause we recommend reading *Defining Death: The Case for Choice*.^5^
   3. Emphasize that choosing a view of death requires scientific (e.g., level of physiologic functioning) and philosophical claims (e.g., determining when a human person has ceased to exist). We observed that participants found it helpful to compare this concept to abortion, where the philosophical concept of a person is mapped onto a biological stage in embryonic development.
   4. Emphasize that a conscience clause does not currently exist in the UDDA. The purpose of the discussion is to be aware of current debate over the medico-legal definition of death.

**Deepening**

During deepening, ask the trainees to apply the new information acquired during the discovery step to their clinical practice. This portion of the de-brief can be used to reflect on the training as a whole. Ask trainees what they learned during the training, how their understanding of brain death has changed, and how this newly acquired knowledge will impact their work in the ICU.

References

1. Russell JA, Epstein LG, Greer DM, Kirschen M, Rubin MA, Lewis A. Brain death, the determination of brain death, and member guidance for brain death accommodation requests: AAN position statement. Neurology. 2019;92(5):228-232. <https://doi.org/10.1212/WNL.0000000000006750>

2. Greer DM, Shemie SD, Lewis A, et al. Determination of brain death/death by neurologic criteria: the world brain death project. JAMA. 2020;324(11):1078. <https://doi.org/10.1001/jama.2020.11586>

3. Morrison WE, Kirschen MP. A taxonomy of objections to brain death determination. Neurocrit Care. 2022;37(2):369-371. <https://doi.org/10.1007/s12028-022-01580-6>

4. Veatch RM. Controversies in defining death: a case for choice. Theor Med Bioeth. 2019;40(5):381-401. <https://doi.org/10.1007/s11017-019-09505-9>

5. Veatch RM, Ross LF. *Defining Death: The Case for Choice*. Georgetown University Press; 2016.
