# Supplementary material for: A Simulation to Improve Understanding and Communication of Ethical Dilemmas That Surround Brain Death
Source: MedEdPORTAL. 2024 Sep 26;20:11444. doi: 10.15766/mep_2374-8265.11444 (PMC11424717; doi:10.15766/mep_2374-8265.11444)
Supplement: Supplementary file 1 — Prebrief Instructions and Presentation.pptxStandardized Patient Case Development Tool.docxSimulation Case.docxWBUH Checklist for Determining Brain Death.docxInstructions for Debrief.docxQuestionnaire.docx [file mep_2374-8265.11444-s001.zip › A. Prebrief Instructions and Presentation.pptx]

## Slide 1
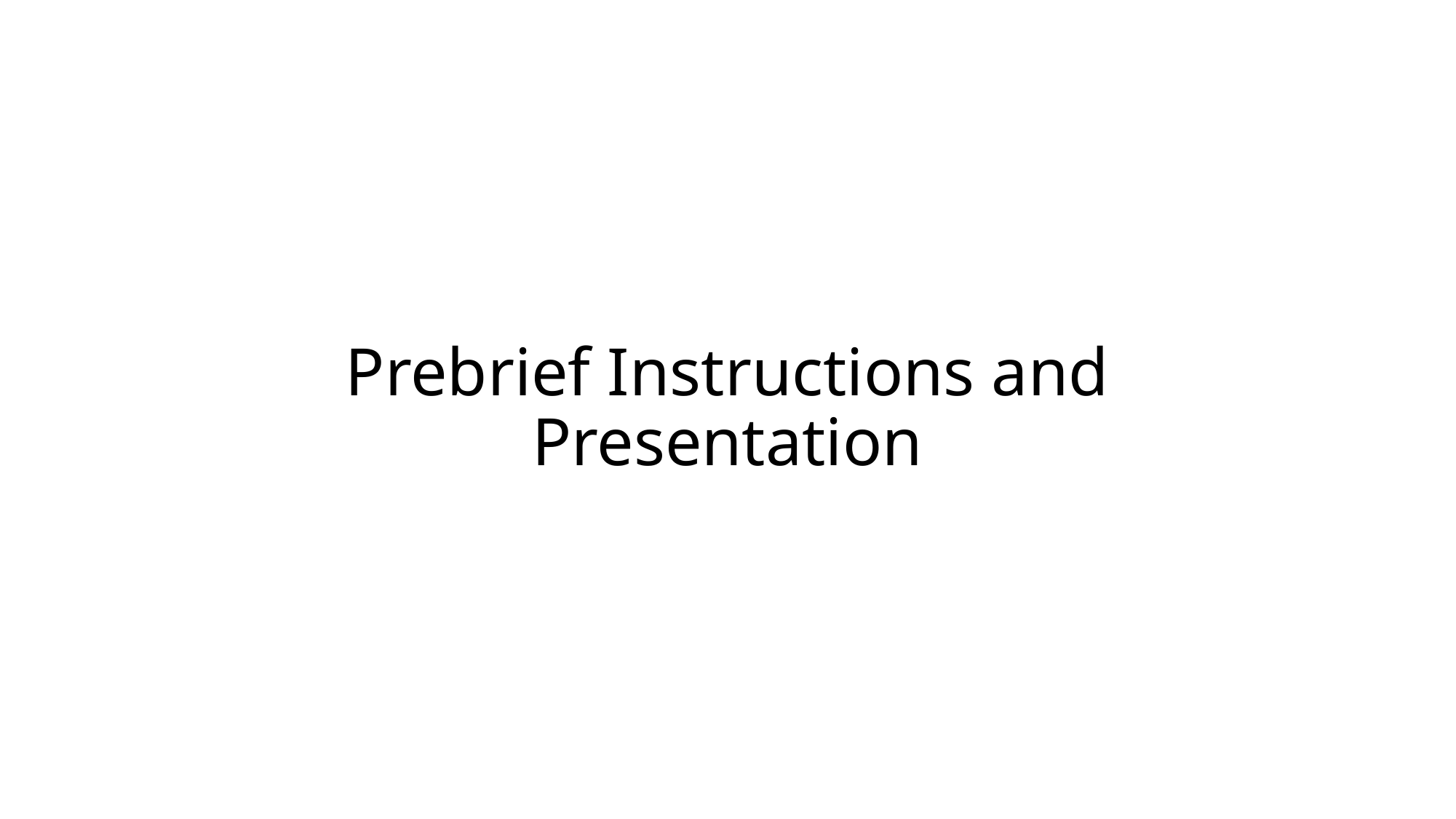

# Prebrief Instructions and Presentation

## Slide 2
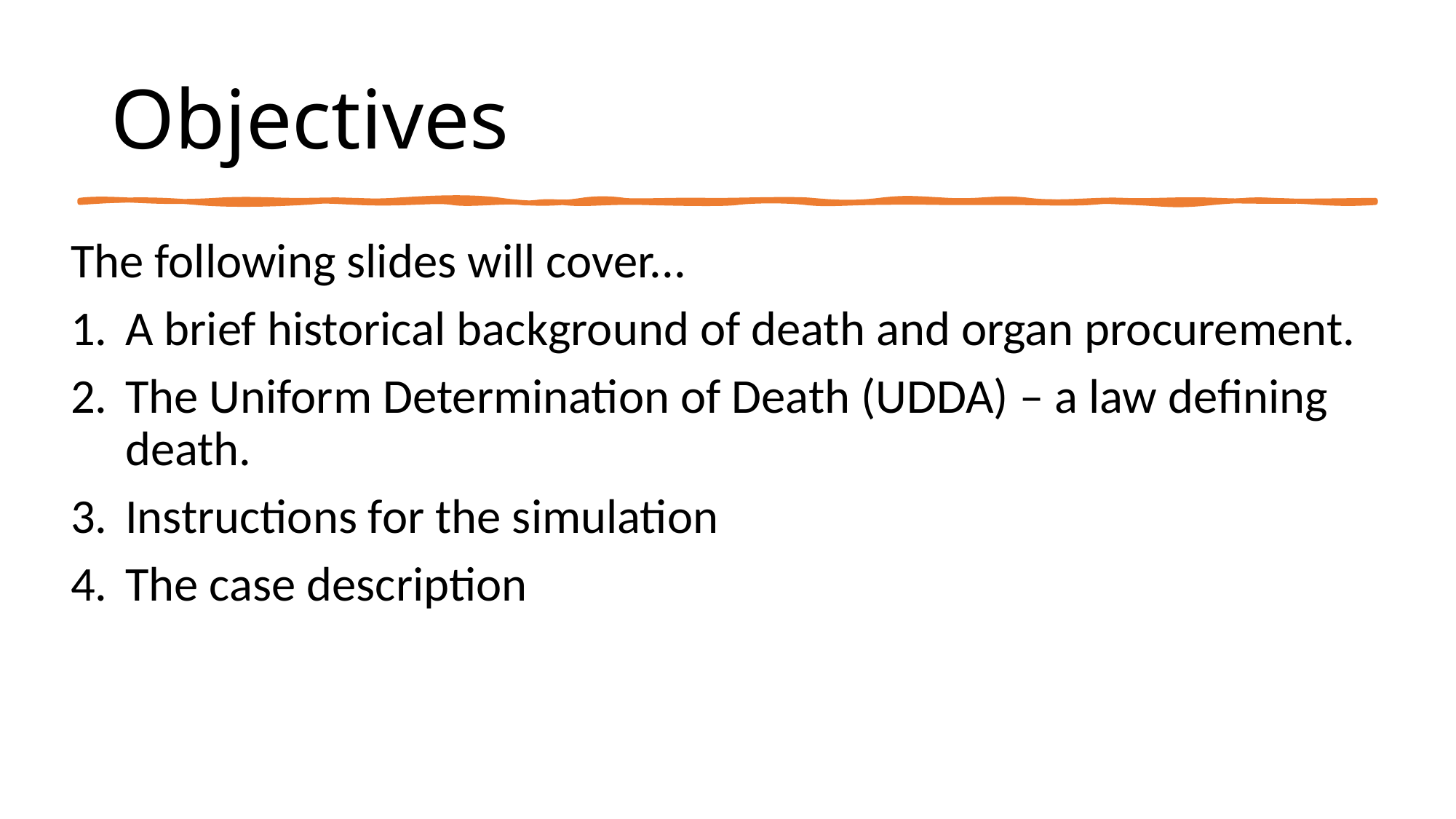

# Objectives
The following slides will cover...
A brief historical background of death and organ procurement.
The Uniform Determination of Death (UDDA) – a law defining death.
Instructions for the simulation
The case description

## Slide 3
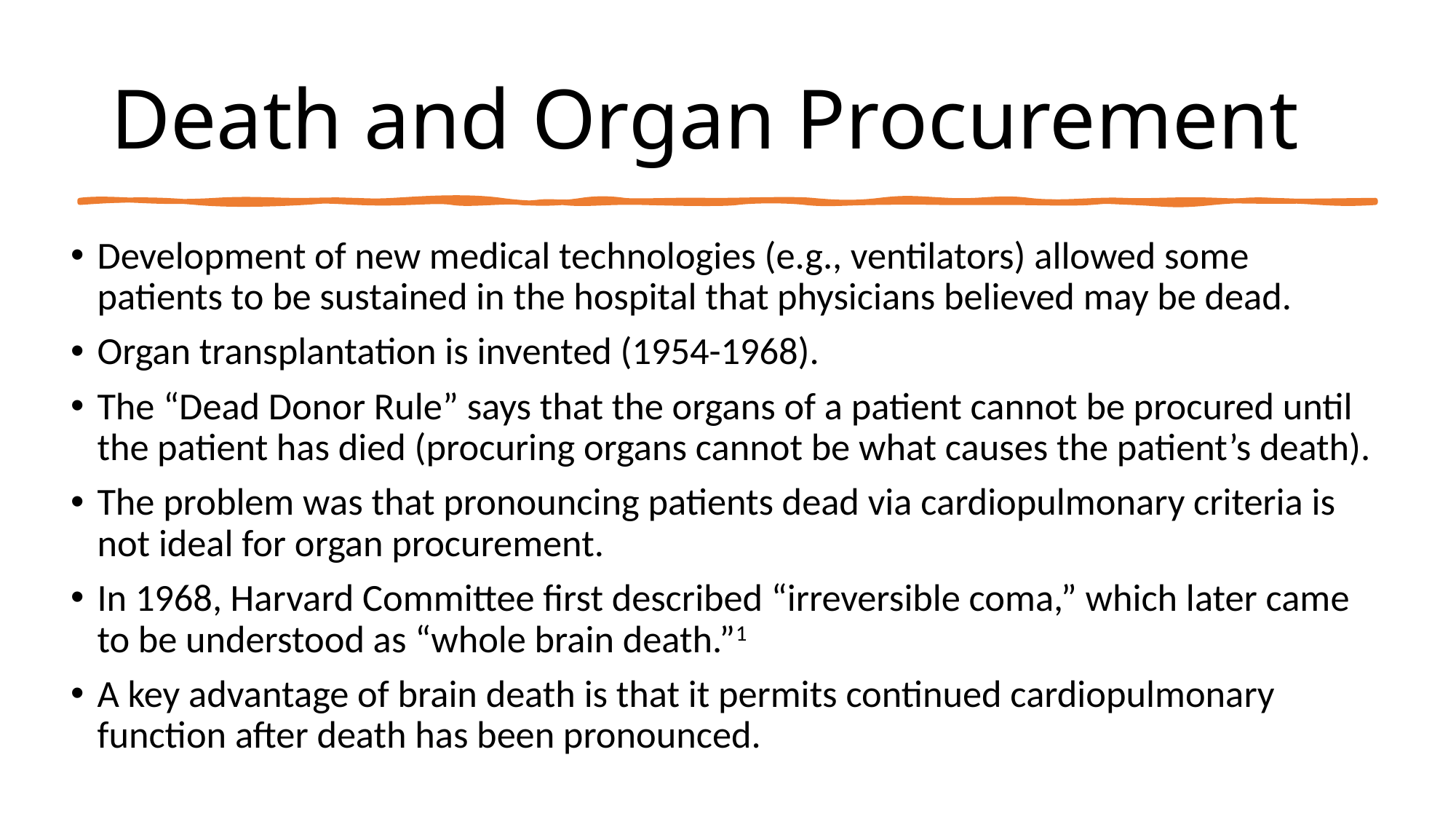

# Death and Organ Procurement
Development of new medical technologies (e.g., ventilators) allowed some patients to be sustained in the hospital that physicians believed may be dead.
Organ transplantation is invented (1954-1968).
The “Dead Donor Rule” says that the organs of a patient cannot be procured until the patient has died (procuring organs cannot be what causes the patient’s death).
The problem was that pronouncing patients dead via cardiopulmonary criteria is not ideal for organ procurement.
In 1968, Harvard Committee first described “irreversible coma,” which later came to be understood as “whole brain death.”1
A key advantage of brain death is that it permits continued cardiopulmonary function after death has been pronounced.

## Slide 4
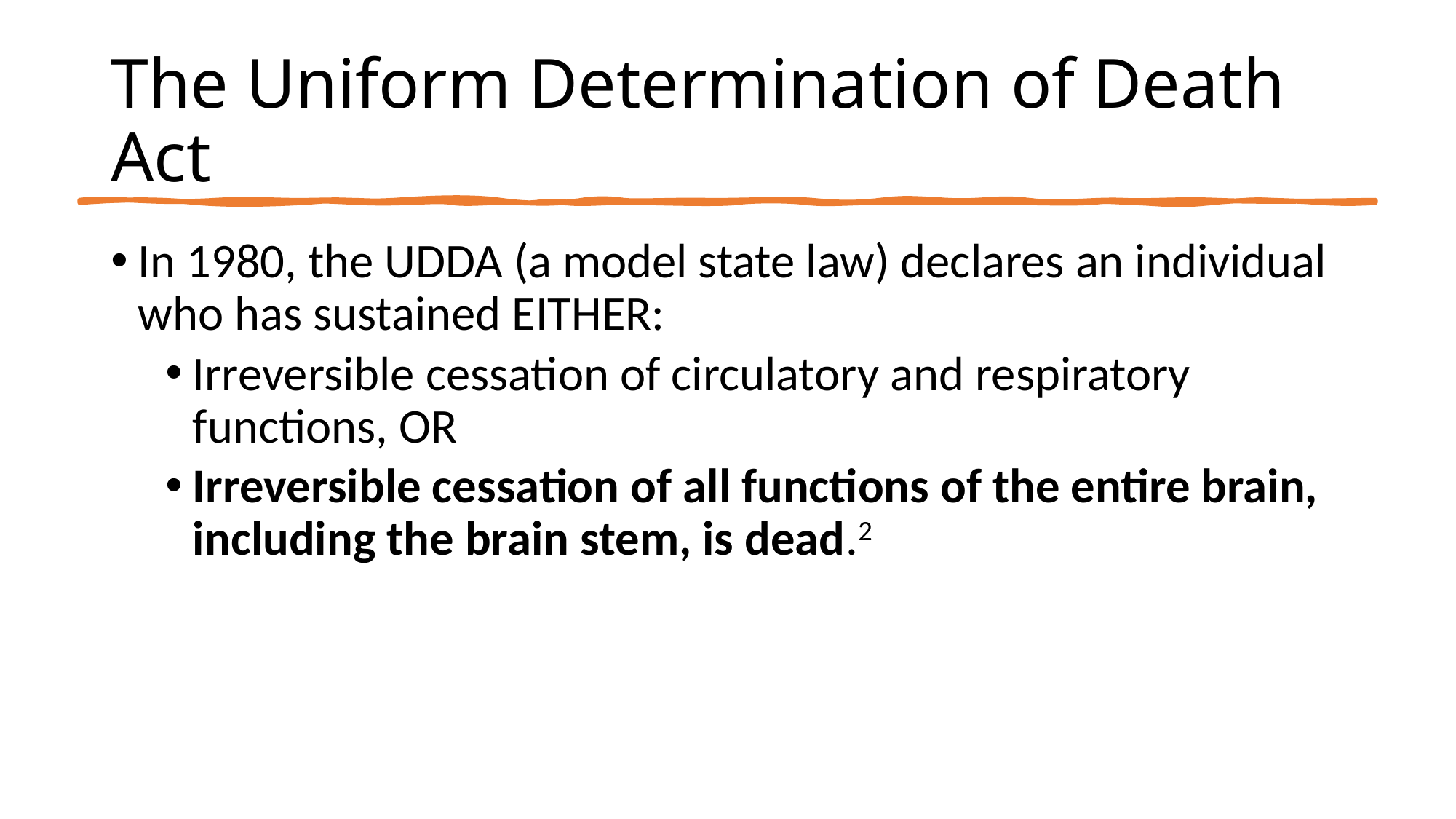

# The Uniform Determination of Death Act
In 1980, the UDDA (a model state law) declares an individual who has sustained EITHER:
Irreversible cessation of circulatory and respiratory functions, OR
Irreversible cessation of all functions of the entire brain, including the brain stem, is dead.2

## Slide 5
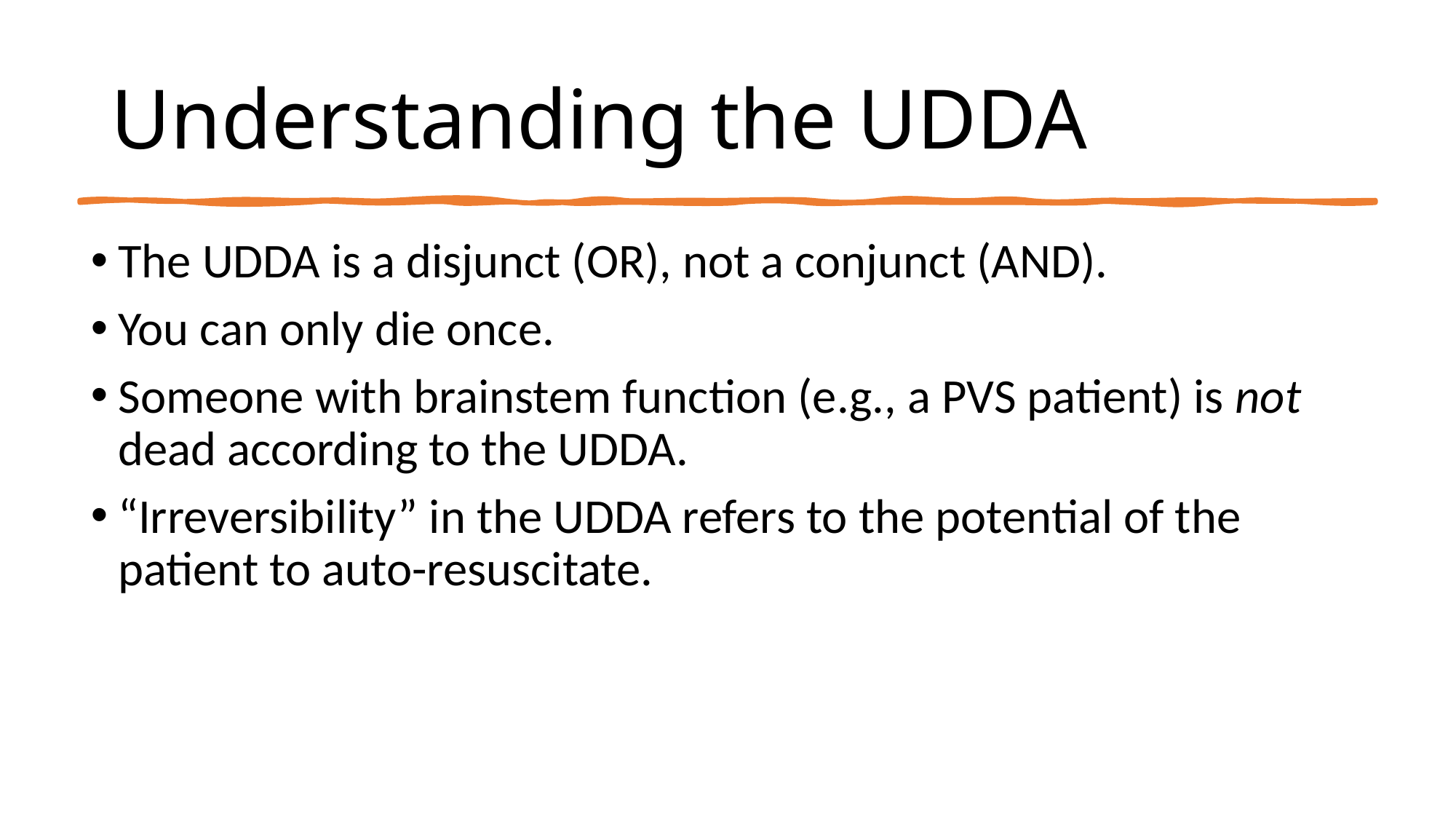

# Understanding the UDDA
The UDDA is a disjunct (OR), not a conjunct (AND).
You can only die once.
Someone with brainstem function (e.g., a PVS patient) is not dead according to the UDDA.
“Irreversibility” in the UDDA refers to the potential of the patient to auto-resuscitate.

## Slide 6
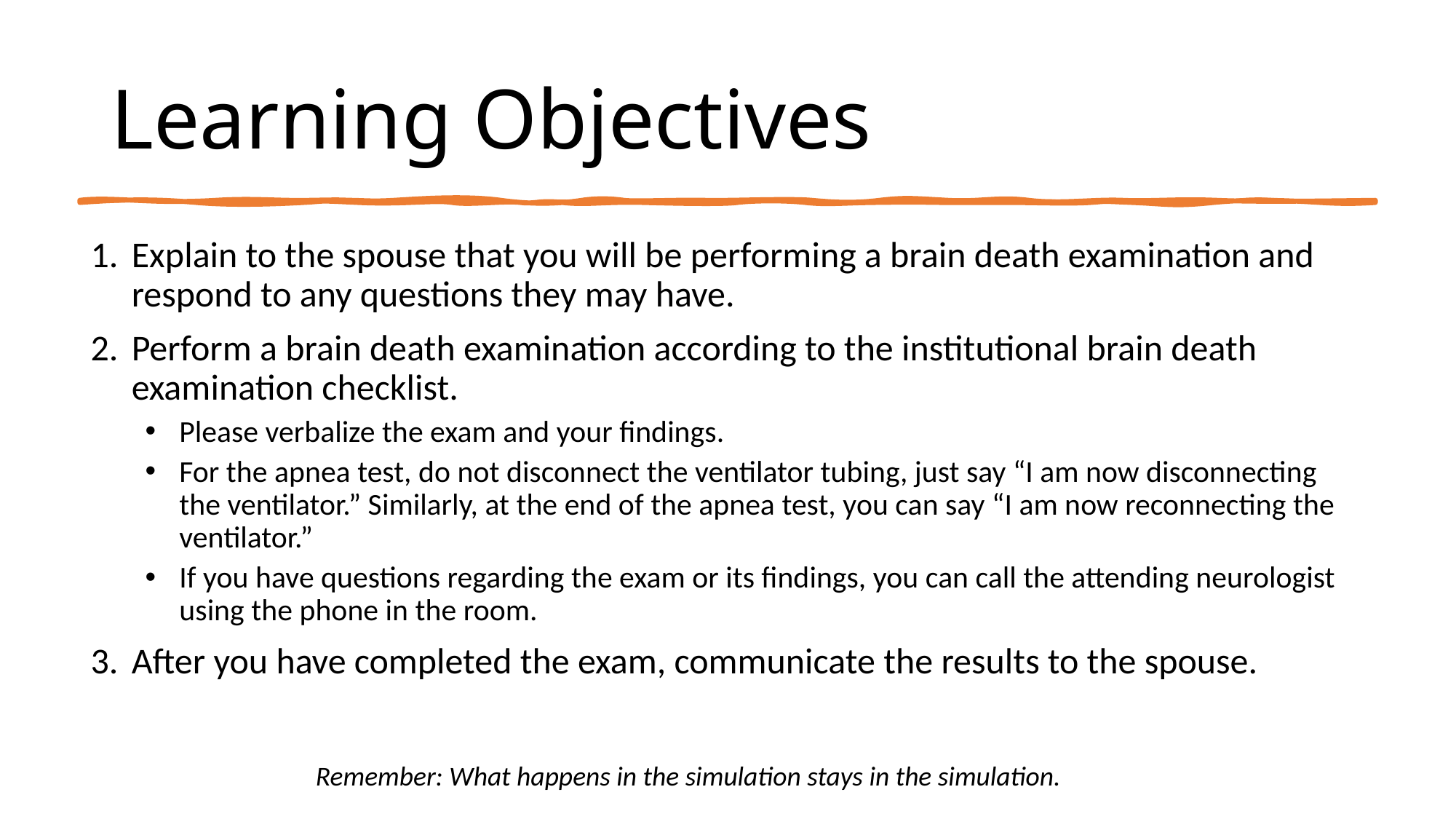

# Learning Objectives
Explain to the spouse that you will be performing a brain death examination and respond to any questions they may have.
Perform a brain death examination according to the institutional brain death examination checklist.
Please verbalize the exam and your findings.
For the apnea test, do not disconnect the ventilator tubing, just say “I am now disconnecting the ventilator.” Similarly, at the end of the apnea test, you can say “I am now reconnecting the ventilator.”
If you have questions regarding the exam or its findings, you can call the attending neurologist using the phone in the room.
After you have completed the exam, communicate the results to the spouse.
Remember: What happens in the simulation stays in the simulation.

## Slide 7
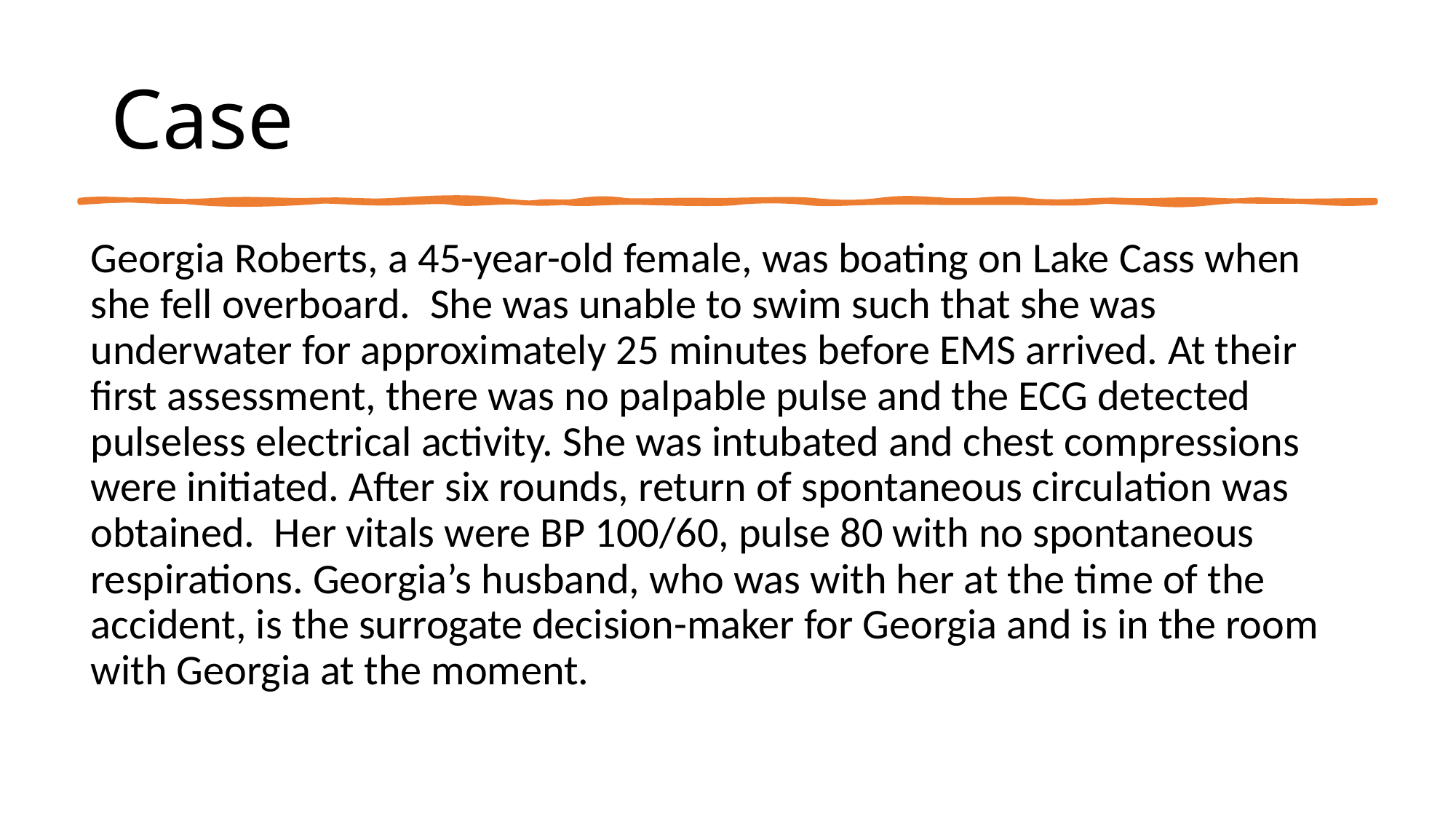

# Case
Georgia Roberts, a 45-year-old female, was boating on Lake Cass when she fell overboard.  She was unable to swim such that she was underwater for approximately 25 minutes before EMS arrived. At their first assessment, there was no palpable pulse and the ECG detected pulseless electrical activity. She was intubated and chest compressions were initiated. After six rounds, return of spontaneous circulation was obtained.  Her vitals were BP 100/60, pulse 80 with no spontaneous respirations. Georgia’s husband, who was with her at the time of the accident, is the surrogate decision-maker for Georgia and is in the room with Georgia at the moment.
