# Supplementary material for: A Simulation to Improve Understanding and Communication of Ethical Dilemmas That Surround Brain Death
Source: MedEdPORTAL. 2024 Sep 26;20:11444. doi: 10.15766/mep_2374-8265.11444 (PMC11424717; doi:10.15766/mep_2374-8265.11444)
Supplement: Supplementary file 1 — Prebrief Instructions and Presentation.pptxStandardized Patient Case Development Tool.docxSimulation Case.docxWBUH Checklist for Determining Brain Death.docxInstructions for Debrief.docxQuestionnaire.docx [file mep_2374-8265.11444-s001.zip › B. Standardized Patient Case Development Tool.docx]

Appendix B: Standardized Patient Case Development Tool

Date: 1/22/2024

Primary Case Author: Nicholas Ludka

Secondary Case Author: Ngan Nguyen Ph.D., Daniel Menkes M.D., M.H.S.A., Abram Brummett Ph.D., HEC-C

Standardized Patient Educator: Nicholas Ludka

Name of Case: Managing Ethical Dilemmas in a Case of Simulated Brain Death

Name of Educational and/or Assessment Activity: A Simulation to Improve Understanding and Communication of Ethical Dilemmas That Surround Brain Death

Patient Name: Georgia (George) Roberts; name will depend on the chosen sex of the patient

Chief Complaint: The patient (SP’s spouse) is unresponsive after drowning in a boating accident.

Most Likely Diagnosis and Differential With Rationale From History and/or Physical Exam: Brain death – patient history and physical examination

Challenge Question: Explain, perform, and discuss the result of the brain death examination with the patient’s spouse using appropriate language.

Domains: Check all that apply

⛝ Professionalism

⛝ Communication and Interpersonal Skills

- Medical History

⛝ Physical Exam

⛝ Shared Decision-Making

⛝ Patient Education

- Clinical Reasoning
- Documentation
- Handoff
- Presentation
- Other:

Type and Level of Learner: Medical students – 3^rd^ and 4^th^ year; Residents - 1^st^ to 3^rd^ year; Pulmonary and critical care fellows – 1^st^ and 2^nd^ year.

Case Objectives: By the end of the simulation, trainees will be able to:

1. Discuss the brain death examination with the spouse.
2. Respond appropriately to the spouse’s objection to the brain death examination.
3. Perform a brain death examination according to the institutional protocol.
4. Explain the exam findings to the spouse.
5. Respond appropriately to the spouse’s philosophical objections to brain death and wishes to continue life-sustaining therapies.

| SETTING: outpatient, in patient, ED, home, nursing home, rehab, group, etc. | Intensive care unit |
| --- | --- |
| SPOUSE PROFILE: Information about the “spouse” that helps select an SP and helps the learner get an understanding of them as a person. SP will know more information about the spouse than learner will ever ask but allows SP to portray a fully developed personality. If none of the items below are particulars for the case, please write “all may be used.” | |
| Age range | 25-65 (confederate from research team was 26) |
| Religious/spiritual background | All may be used |
| Sex (e.g., male, female, intersex, transwoman, transman) | All may be used |
| Sexual orientation (e.g., heterosexual, lesbian, gay, bisexual, pansexual, queer, asexual) | All may be used |
| Gender expression (e.g., man, woman, genderqueer) | All may be used |
| Race and ethnicity | All may be used |
| Physical description (e.g., BMI, height range) | All may be used |
| Physical limitations | None |
| Spouse appearance (e.g., disheveled, hospital gown, business casual, casual) | Casual |
| Moulage + location (e.g., none, bruises, scars, body piercing, tattoos) | None |
| Affect (e.g., pleasant, cooperative) | Occasionally shows signs of sadness but mostly optimistic that the patient is still alive. Curious about what additional medications or interventions can be offered to their spouse to help them recover. Overall is pleasant but can become confrontational (see case information). |
| Family group (e.g., who is family, who they live with) | Lived alone with spouse. No children. Have been married for five years. |
| Education | College education |
| Level of brain death literacy | Little to none; they have heard of the term “brain death” in the news but does not know what it means. |
| Employment, if any - present and past, noting any current stresses | Works as an accountant for a small local CPA firm. |
| Home/homeless - type of dwelling, number of stories, owned or rented | Currently renting a ranch-style house with their spouse. |
| Financial situation - any current stresses | Not under financial stress. |
| Insurance status (e.g., un/under/insured, public/private, HMO/PPO) | Private insurance |
| Habits (i.e., diet, exercise, caffeine, smoking, alcohol, drugs) | Drinks alcohol on occasion, no smoking or drug use. |

| CASE INFORMATION | |
| --- | --- |
| Chief Concern: What the spouse will say when greeted by the trainee. | “These have been a few tough days but I think we’re through the worst of it. I’m just hoping you can give me some good news. I know George/Georgia is a fighter and will get through this.” |
| Additional Concerns: Other concerns the spouse has during the encounter. | General Concerns  **-** If medical jargon is used, the spouse should ask for clarification. For example, if the trainee says “apnea test” the spouse may ask “apnea; what does that mean?”  **-** The spouse should pay close attention to any mixed messages being communicated by the trainee. We encountered this many times in our simulations; a trainee would say that the patient was “brain dead” and that they would be withdrawing “life support.” This prompted the spouse to reply, “Life support? I thought you just said that they are dead? So they are alive, right?”  Concerns during pre-examination discussion with trainee  **-** The spouse should repeatedly assert themselves as the patient’s surrogate decision maker. They have been married to their spouse for many years and have been legally appointed as their health care agent. They take this responsibility very seriously, and repeatedly state that “I want to do what is best for my partner.”  **-** There should be a strong refusal to consent to the brain death examination. The spouse should repeatedly state that their partner would not want a brain death examination, saying something like “I am not going to let you touch my partner without my approval. They would never want something like this done.”  **-** If the trainee says the words “brain death” during the pre-examination discussion, the spouse should become increasingly worried. They may cite recent cases of brain death in the news (e.g., Archie Battersbee or Anne Heche), and how the doctors in those cases killed the patients by withdrawing the ventilators.  **-** Emphasis should be placed on the vital signs being displayed on the monitor. The spouse and the patient have a circulatory view of death, and as such, the heartbeat and lungs are the critical functions that must be lost before death can be declared. The spouse should make references to the stable blood pressure, heart rate, and blood oxygenation, demonstrating that their partner is still alive according to their beliefs.  - Depending on the direction of the conversation, the spouse may also express their worries about the brain death examination and organ procurement. They may say something like, “you only want to declare death so that you can take their organs.” This should prompt the trainee to explain the relationship between the medical team and the organ procurement organization.  Concerns during brain death examination  **-** The spouse may sit quietly at the bedside while the trainee conducts the brain death examination. However, they may interject with questions such as “what does that mean?” or “doesn’t that hurt them?” Given the time constraints to deliver the session within an hour, the spouse may be cut off by the facilitator so that the trainee can finish the exam in a timely manner.  Concerns during pre-examination discussion with trainee  **-** If the trainee says that the patient is “brain dead” the spouse may concede that they are brain dead, but that this is not equivalent to death. If the trainee says that the patient is “dead” the spouse should repeat their argument that was mentioned in the pre-examination discussion (i.e., the patient is alive so long as their heart and lungs are functioning).  **-** The spouse should demand that all life-sustaining therapies be continued and that their partner have a full-code designation. The spouse believes that their partner is alive, and as such, wants to give them every chance to recover. They may say something like “as a spouse, is it not my duty to do everything I possibly can to help my partner?”  **-** In some simulations, the trainee explained to the spouse that brain death is equivalent to death because the patient no longer has an acceptable quality of life. If this, or related statements, are made by the trainee, the spouse should reply “How do you have the authority to determine what is an acceptable quality of life? Shouldn’t I be making that determination? After all, you don’t even know my partner.” This should prompt the trainee to contemplate why brain death is equivalent to death, which is covered afterward in the debrief.  **-** The spouse should be apprehensive of the finality of the brain death diagnosis. In other words, the spouse should have a strong belief that the patient’s condition is reversible. Therefore, not only would removing the patient from the ventilator constitute murder in the eyes of the spouse, it would also indicate the medical team giving up on a patient who has a chance at a meaningful recovery. |
| THE SPOUSE’S STORY:  The personal context should be able to answer questions concerning the broader personal/psychosocial context of the patient’s admission to the intensive care unit, especially the patient’s beliefs/attributions.  The emotional context should be able to ask how are you doing with this, how does this make you feel, how has this affected you emotionally? IMPACT: How has this affected your life? How has this been for your family? | “We were out at the lake with some friends for the weekend. A few of us piled in the boat for a ride around the lake. The weather couldn’t have been more perfect. Between that high activity on the lake and the wind, it was a bumpy ride with some large waves. George/Georgia was standing on the side of the boat when we hit a large wave. They stumbled backward and grabbed onto the railing, but all of a sudden, we hit another wave and before we knew it George/Georgia was thrown over the side of the boat. They tried to swim but the waves were just too big (becomes withdrawn)... I’m just glad the emergency team got their when they did and were able to get the heartbeat back.  NOTE: The trainee will get a brief description of the circumstances of the accident before beginning the simulation. Therefore, don’t expect the trainee to ask further questions regarding the circumstances of the accident. If they do, use the above story. If not, you can begin with the story below.  I think that the worst is behind us. That first day was awful. The first time I saw George/Georgia hooked up to all these machines and monitors is something I’ll never forget. I called all of our family and friends to let them know of the circumstances and they’ve been so supportive. I’ve got a great network that’s been really helpful. Now that we’re through that initial shock I think I’m feeling a little bit better. I feel like I’m finally at a place where I can start to look ahead to what’s next. When can he/she get off the ventilator? What does the new normal look like for us? Does he/she need surgeries, different medications? There was the initial phase of, “let’s make sure George/Georgia is alive” and now I think we’ve transitioned into the next phase of, “what can we do to make George/Georgia better?” I know that George/Georgia is a fighter. If there is anybody in this world that is capable of recovering from such an injury it’s him/her. Shortly after we got married, we signed some form of agreement that allows each of us to make medical decisions for the other. I promised him that, heaven forbid anything were to happen to him, I would do everything I could to make sure he survives.” |
