# Supplementary material for: A Simulation to Improve Understanding and Communication of Ethical Dilemmas That Surround Brain Death
Source: MedEdPORTAL. 2024 Sep 26;20:11444. doi: 10.15766/mep_2374-8265.11444 (PMC11424717; doi:10.15766/mep_2374-8265.11444)
Supplement: Supplementary file 1 — Prebrief Instructions and Presentation.pptxStandardized Patient Case Development Tool.docxSimulation Case.docxWBUH Checklist for Determining Brain Death.docxInstructions for Debrief.docxQuestionnaire.docx [file mep_2374-8265.11444-s001.zip › F. Questionnaire.docx]

**Appendix F. Questionnaire**

**Demographics**

Q1: Please select your gender.

Male, Female, Other (write in), Prefer not to answer

Q2: Please select your program.

Medical student, Transitional year, Preliminary year, Neurology residency, Pediatrics residency, Neurosurgery residency, General surgery residency, Internal medicine residency, emergency medicine residency, anesthesiology residency, Pulmonary and critical care fellowship, Surgical critical care fellowship, Other (write in)

Q3: Please select your year

PGY-1, PGY-2, PGY-3, PGY-4, PGY-5, PGY-6, F1, F2, F3, Other (write in)

Q4: Before coming to the simulation, I had performed a brain death examination

Yes, No

**Understanding**

Q5: I can describe the higher-brain and circulatory views of death.

1- Strongly Disagree, 2-Disagree, 3- Neither Agree nor Disagree, 4- Agree, 5- Strongly Agree

Q6: I understand the relationship between the dead donor rule and the two methods of organ procurement.

1- Strongly Disagree, 2-Disagree, 3- Neither Agree nor Disagree, 4- Agree, 5- Strongly Agree

Q7: I understand how death is determined in the Uniform Determination of Death Act.

1- Strongly Disagree, 2-Disagree, 3- Neither Agree nor Disagree, 4- Agree, 5- Strongly Agree

**Attitudes**

Q8: I appreciate that non-standard views of death are not based on false clinical beliefs but represent different philosophical views of death.

1- Strongly Disagree, 2-Disagree, 3- Neither Agree nor Disagree, 4- Agree, 5- Strongly Agree

Q6: I am frustrated with families who hold a circulatory view of death.

1- Strongly Disagree, 2-Disagree, 3- Neither Agree nor Disagree, 4- Agree, 5- Strongly Agree

Q7: I am frustrated with families who hold a high-brain view of death.

1- Strongly Disagree, 2-Disagree, 3- Neither Agree nor Disagree, 4- Agree, 5- Strongly Agree

**Confidence**

Q8: I am confident in my ability to perform a clinical examination to determine brain death in adults.

1- Strongly Disagree, 2-Disagree, 3- Neither Agree nor Disagree, 4- Agree, 5- Strongly Agree

Q9: I am confident in my ability to respond appropriately to common ethical issues that arise around brain death.

1- Strongly Disagree, 2-Disagree, 3- Neither Agree nor Disagree, 4- Agree, 5- Strongly Agree

Q10: I am confident in my ability to communicate appropriately with families about brain death.

1- Strongly Disagree, 2-Disagree, 3- Neither Agree nor Disagree, 4- Agree, 5- Strongly Agree

**Feedback (open-ended response)**

Q11: Please list area(s) of improvement

Q12: What did you like most about the training?
