# Supplementary material for: A Simulation to Improve Understanding and Communication of Ethical Dilemmas That Surround Brain Death
Source: MedEdPORTAL. 2024 Sep 26;20:11444. doi: 10.15766/mep_2374-8265.11444 (PMC11424717; doi:10.15766/mep_2374-8265.11444)
Supplement: Supplementary file 1 — Prebrief Instructions and Presentation.pptxStandardized Patient Case Development Tool.docxSimulation Case.docxWBUH Checklist for Determining Brain Death.docxInstructions for Debrief.docxQuestionnaire.docx [file mep_2374-8265.11444-s001.zip › C. Simulation Case.docx]

**Appendix C. Simulation Case**

| **SIMULATION CASE TITLE:** Managing Ethical Dilemmas in a Case of Simulated Brain Death  **AUTHORS:** Nicholas Ludka, Ngan Nguyen Ph.D., Daniel Menkes M.D., M.H.S.A., Abram Brummett Ph.D., HEC-C  **LEARNER AUDIENCE:** Medical students, residents, fellows | |
| --- | --- |
| **PATIENT NAME:** Georgia Roberts  **PATIENT AGE:** 45  **CHIEF COMPLAINT:** Anoxic brain injury  **PHYSICAL SETTING:** Medical ICU in a tertiary care center | |
|  | |
| **Brief Narrative Description of Case** | The case involves a patient who has experienced an anoxic brain injury and is currently unresponsive. In order to determine the next best steps, the medical team needs to determine if the patient is brain dead. A medical trainee is tasked with performing a brain death examination on the patient. Before doing so, they must discuss the examination with the spouse, and after their exam, communicate their findings. Throughout the interaction the trainee is met with objections and challenges from the patient’s spouse. |
| **Primary Learning Objectives** | - Discuss the brain death examination with the spouse - Respond appropriately to the spouse’s objection to the brain death examination - Perform a brain death examination according to the institutional protocol - Explain the exam findings to the spouse - Respond appropriately to the spouse’s philosophical objections to brain death and wishes to continue life-sustaining therapies |
| **Critical Actions** | *Critical actions for examination*   - Check that the core body temperature is greater than or equal to 35° C (95°F) - Confirm absence of spontaneous or induced cerebral motor response to painful stimulation within the cranial nerve distribution. - Confirm decerebrate and decorticate responses or seizures are absent. - Confirm absence of pupillary response to a bright light in both eyes. - Confirm the absence of corneal reflexes and spontaneous eye movements. - Confirm absence of eye movements in response to vestibular stimulation by cold caloric testing. - Confirm absence of eye movements in response to oculocephalic reflex testing. - Check absence of gag reflex in response to stimulation of posterior pharynx. - Check absence of cough reflex in response to deep bronchial suctioning. - Confirm absence of spontaneous respiration at pCO2 greater than or equal to 60 mmHg and an increase in pCO2 of greater than or equal to 20mmHg above pre-apnea test level. Evident respiratory acidosis at the completion of the apnea test.   *Critical actions for communication*   - Use the term “death” instead of “brain death.” - Assess the spouse’s understanding of the patient’s diagnosis and prognosis - Avoid using language that suggests the patient is alive, (e.g., describing a brain-dead patient on life support). - Explain the relationship between the clinical team and the organ procurement organization (OPO), (i.e., the OPO is not involved with death determination. - Omit medical jargon) - Emphasize that recovery is impossible. - Provide emotional support to the spouse. - Give the spouse opportunities to ask questions. |
| **Learner Preparation or Prework** | There was no required advanced preparation. Any relevant information was covered during the prebrief didactic session. |

| Initial Presentation | | | |
| --- | --- | --- | --- |
| **Initial Vital Signs** | HR 75, BP 120/80, RR 20 (maintained by ventilator), O_2_ saturation 99%, Temp 98.3°F | | |
| **Overall Setting and Appearance** | The patient is in a medical ICU room and is connected to a ventilator. | | |
| **Standardized Participants (and Their Roles in the Room at Case Start)** | The patient’s spouse is sitting at the bedside. They are overwhelmed by the current situation, but optimistic that their spouse will recover from their injuries. They repeatedly make note of the vital signs on the monitor across the room, emphasizing that their spouse is doing well considering the circumstances. | | |
| **HPI** | The following case was presented to the trainee prior to the start of the simulation:  Georgia Roberts, a 45-year-old female, was boating on Lake Cass when she fell overboard.  She was unable to swim such that she was underwater for approximately 25 minutes before EMS arrived.   At their first assessment, there was no palpable pulse and the ECG detected pulseless electrical activity. She was intubated and chest compressions were initiated. After six rounds of CPR, return of spontaneous circulation (ROSC) was obtained.  Her vitals were BP 100/60, pulse 80 with no spontaneous respirations. | | |
| **Past Medical/Surgical History** | **Medications** | **Allergies** | **Family History** |
| None | None | None | None |
| **Physical Examination** | | | |
| **General** | No signs of wakefulness or awareness | | |
| **HEENT** | No scleral icterus, no conjunctival injection, normocephalic/atraumatic, normal dentition, pink oral mucosa, pharynx without exudate, oral intubation | | |
| **Neck** | Supple, no masses, trachea midline, no adenopathy | | |
| **Lungs** | Clear lungs bilaterally, no spontaneous respiratory drive. | | |
| **Cardiovascular** | Regular rate and rhythm, no murmurs, no edema, brisk carotid upstroke, absence or carotid bruit | | |
| **Abdomen** | Soft, no masses, normoactive bowel sounds | | |
| **Neurological** | No response to painful stimuli. No eye opening. Pupils are non-reactive to light. No spontaneous eye movements. Absence of oculocephalic and vestibulo-ocular reflex. No gag or cough reflex. Absence of posturing (decerebrate or decorticate). | | |
| **Skin** | Normal coloration, no rashes, no lesions | | |
| **GU** | No discharge, masses, or induration | | |
| **Psychiatric** | N/A | | |

| Instructor Notes - Changes and CASE Branch Points. | | |
| --- | --- | --- |
| **Intervention / Time Point** | **Change in Case** | **Additional Information** |
| Spouse refuses to consent to brain death examination. |  | If it appears that the trainee is stuck on what to do and too much time has elapsed, the facilitator announces over the loud speaker, "Pause simulation, please proceed with the brain death examination.” |
| Spouse repeatedly asks questions to the trainee as they are performing the brain death examination. For example, “why are you doing that?” or “you’re hurting them!” while they are testing for reaction to painful stimulus. | If too much time is being spent on questioning such that the simulation will take too long to complete, the facilitator will speak through the headset to tell the spouse to stop asking questions or keep their remarks short |  |
| Trainee verbalizes that they are disconnecting the ventilator for the apnea test | Simulation technician stops respirations, slowly decreases the O_2_ saturation, and drops the respiratory rate to 0. |  |
| Trainee makes reference to an unacceptable quality of life during the discussion of the exam findings. | Spouse becomes visibly upset and more defensive, arguing that the physician has no right to assert their own view of what is an acceptable quality of life. |  |

**Ideal Scenario Flow**

The trainee enters the patient's room, where they find the patient's spouse sitting at the bedside. The trainee introduces themselves as one of the physicians taking care of the patient and asks the patient's spouse for their understanding of the patient's current situation. The trainee says that an examination is needed in order to get a better understanding of the patient’s current neurological condition. The trainee requests that the spouse exit the room before they conduct the exam. After the spouse exits the room, the trainee proceeds to work through the provided checklist for determining brain death. Before each step, they announce what maneuver they will be performing and the reaction, or lack thereof, that is demonstrated by the patient. Given the exam findings, the trainee determines that the patient is brain dead. After the trainee completes the examination, they invite the spouse back into the room. The learner then sits next to the patient's spouse and asks for the spouse's permission to discuss the examination findings. Without use of medical jargon or misleading language, the trainee tells the spouse that the patient has died. The trainee then gives ample time for the spouse to process this information, and responds to any questions the spouse may have. The learner explains the irreversibility of brain death, and that the vital signs displayed on the monitor represent the patient's heartbeat and breathing that are being sustained by medical intervention. The simulation concludes with the trainee offering their condolences to the spouse and giving them further time to process the information. The trainee may mention that they will come back shortly to discuss what will happen next.

**Anticipated Management Mistakes**

1. *Confusion with consent requirement for examination*: We found that all of the trainee that participated in the simulation were unsure of whether consent was required for a brain death examination. In the debrief, we made sure to explicitly state that consent is NOT required for a brain death examination and gave the rationale for why. We also offered solutions if it ever became an intractable problem (e.g., consult the ethics and legal services).
2. *Use of misleading language*: Some trainee started their post-examination discussion by saying that the patient was “brain dead” instead of saying “dead.” We thought this was a natural response coming from medical trainees; they are often talking about brain death with *other medical professionals* who understand that brain death and death are one in the same. Yet, this understanding is not necessarily appreciated by non-medical persons. Leading with “brain dead” prompted the spouse to explain that brain death and death are not mutually exclusive (i.e., the patient is alive *despite* being brain dead). Furthermore, some trainee said that they would be withdrawing “life support” or “life-sustaining therapies,” which confused the spouse as to whether the patient was dead.
