# Supplementary material for: A Simulation to Improve Understanding and Communication of Ethical Dilemmas That Surround Brain Death
Source: MedEdPORTAL. 2024 Sep 26;20:11444. doi: 10.15766/mep_2374-8265.11444 (PMC11424717; doi:10.15766/mep_2374-8265.11444)
Supplement: Supplementary file 1 — Prebrief Instructions and Presentation.pptxStandardized Patient Case Development Tool.docxSimulation Case.docxWBUH Checklist for Determining Brain Death.docxInstructions for Debrief.docxQuestionnaire.docx [file mep_2374-8265.11444-s001.zip › _Educational Summary Report_11444.pdf]

MedEdPORTAL® The AAMC Journal of Teaching and Learning Resources

Original Publication Open Access

A Simulation to Improve Understanding and Communication of Ethical Dilemmas That Surround Brain Death

Nicholas Ludka*, Ngan Nguyen, PhD, Daniel Menkes, MD, MHSA, Abram Brummett, PhD, HEC-C

*Corresponding author: nludka@oakland.edu

Abstract

Introduction: Providers across multiple specialties may be called upon to perform brain death assessments at hospitals that lack specialty neurology or critical care services. To address this need, we developed a brain death curriculum involving simulation and group discussion to prepare medical trainees for brain death testing and communication with surrogate decision-makers. **Methods:** A 1-hour session was delivered to trainees rotating through the intensive care unit at William Beaumont University Hospital. One trainee per session participated in a simulation involving a brain-dead patient (SimMan 3G Mannequin) and spouse (confederate) while the remainder of the cohort observed from a separate room. The trainee briefed the spouse about the brain death examination, performed the examination, and communicated their findings. Afterward, the cohort discussed the history, law, and common ethical and communication issues that surround brain death. **Results:** A total of 35 trainees participated from August 2022 to March 2023. After the session, trainees were more comfortable performing brain death testing ($p < .001$), responding to ethical issues ($p < .001$), and communicating with families ($p < .001$). However, the session did not change their frustration with family members who have a circulatory ($p = .72$) or high brain ($p = .52$) view of death. **Discussion:** The simulation had a positive impact on medical trainees' ability to perform brain death testing and their comfort level in discussing complex ethical issues that surround brain death. Our results support continued simulation training for medical trainees to better prepare them for clinical practice.

Keywords
Brain Death, Philosophy, Simulation, Communication Skills, End of Life/Palliative Care, Interdisciplinary Medicine, Critical Care Medicine, Hospice & Palliative Medicine, Case-Based Learning, Clinical Skills Assessment/OSCEs, Quantitative Research

Educational Objectives

By the end of this activity, learners will be able to:

1. Describe the procedure for determining brain death in adults.
2. Describe the two criteria for determining death as outlined in the Uniform Determination of Death Act.
3. Respond appropriately to surrogate decision-makers when common ethical issues arise during brain death discussions.
4. Identify the three main competing philosophical views of death.
5. Articulate consensus positions on common ethical and communication issues that arise around brain death.

Citation:
Ludka N, Nguyen N, Menkes D, Brummett A. A simulation to improve understanding and communication of ethical dilemmas that surround brain death. *MedEdPORTAL*. 2024;20:11444.
https://doi.org/10.15766/mep_2374-8265.11444

Introduction

Brain death determination is one of the most ethically charged situations a provider can encounter. Not only must providers perform an extensive physical examination that tests cranial nerve function, presence of apnea, and loss of consciousness, they must also communicate the outcome to the patient's loved ones.[1] Physicians who perform brain death assessments have been shown to have low self-confidence in these situations where we should want them to be the very confident (i.e., making the critical determination for whether a person is dead or alive).[2-4] The widespread discomfort that surrounds brain death within the medical community, associated with the variability in brain death examination strategies across hospitals, has motivated some to argue that "a significant time investment must be made by clinicians at all hospitals to champion updating practices to meet accepted standards of care and to help train clinicians in the most

Copyright © 2024 Ludka et al. This is an open-access publication distributed under the terms of the Creative Commons Attribution license.

modern techniques and approaches."[5] Others have highlighted the dangers of brain death misdiagnosis and the need for competency in both diagnosis and communication of brain death.[6,7] Furthermore, as the American Academy of Neurology only suggests that a physician with specific expertise in brain death be a part of the final brain death diagnosis, it is imperative to provide this training to all interested providers.[8] Notably, fewer than 33% of hospitals require brain death examinations to be performed by a physician with expertise in neurology or neurosurgery.[7] Therefore, physicians outside of neurology, neurosurgery, and critical care medicine may be called upon to perform brain death examinations after their training.

Simulation has emerged as a promising methodology for addressing the knowledge and comfort gap in brain death diagnosis and communication.[2-4,8-11] The simulated environment has three distinct advantages to traditional didactic teaching; it offers a low-stakes environment where trainees can openly explore their communication skills, high-fidelity mannequins provide an opportunity to practice clinical skills, and supervision by experts allows the learner to receive immediate feedback that can be integrated into their clinical practice.

A 2020 *MedEdPORTAL* publication by Morris and colleagues emphasized these critical aspects of simulation education.[12] The innovation was structured in an introduction-simulation-debrief format, where a resident performed a brain death examination on a high-fidelity mannequin followed by a family discussion. After the session, among other findings, residents were more comfortable performing brain death examinations and discussing the diagnosis of brain death with families. Our simulation builds off this important work but differs in many significant ways. Morris and colleague's simulation was more resource intensive, requiring additional personnel (e.g., respiratory therapist, social worker, and chaplain) and 3 more hours of time. While our training was intended for a multidisciplinary cohort of trainees at various levels of training, Morris and colleagues only involved neurology residents. A 90-minute simulation that focused on family communication in the context of coma was published in *MedEdPORTAL* in 2014, but only included medical students.[13] This simulation resolved with the family member withdrawing care, whereas our simulation is intentionally left unresolved between the trainee and the family member. Furthermore, while both our simulation and the Morris simulation address appropriate communication (e.g., saying death instead of brain death), we also reviewed common ethical issues that surround brain death,[14] (e.g., whether surrogate consent for examination is needed, and philosophical disagreements on the very definition of death) that are not found in other simulations.

Methods

Development

The simulation was developed by a clinical ethicist, neurologist, and co-director for the surgery simulation fellowship at William Beaumont University Hospital (WBUH), a 1,110-bed high acuity level 1 trauma center in Royal Oak, Michigan. The simulation occurred in the Eugene and Marcia Applebaum Simulation Learning Institute on the WBUH campus. The session was designed for medical students, residents, and fellows who were rotating through the medical intensive care unit. A group of four to seven trainees participated in each session. Limiting group size encouraged participation during the debrief session. The most productive conversations occurred with four to six trainees. The project did not require institutional review board approval, as it did not constitute human subject research.

Learner Prerequisites

There was no required advanced preparation. Any relevant information for the simulation was provided during the prebrief (Appendix A). The learners were not expected to have performed a brain death examination before the session.

Equipment/Environment

- One simulated ICU room.
- One conference room equipped with live audio and video of the ICU room: If a separate room with audio/visual feed is not available, the rest of the trainees may, if space allows, view the simulation behind a one-way mirror with the facilitator or sit and observe in the periphery of the ICU room while the simulation is taking place. While having physical separation between the simulation and the observers would better approximate a true clinical environment, having the observers in closer proximity does not hinder the simulation from occurring.
- Two-way headset for confederate and facilitator.
- High-fidelity mannequin: This is not required, as a low-fidelity mannequin should mimic brain death (e.g., no pupillary reflex, no withdrawal to painful stimuli) and has been shown to be effective elsewhere.[11] Although a high-fidelity mannequin's chest can rise and fall in accordance with the artificial ventilation, we opine that the absence of this additional feature would not significantly detract from the simulation.
- Ventilator: If a ventilator is not available, the learner in the simulation may simply verbalize to the observer when they wish to disconnect the ventilator. The observer could communicate the results of the apnea test via intercom or printed report.

Copyright © 2024 Ludka et al. This is an open-access publication distributed under the terms of the Creative Commons Attribution license.

- Monitor: The simulation was developed with a monitor that showed oxygen saturation, heart rate, blood pressure, and respiratory rate. These values did not change throughout the simulation, save for the respiratory rate and oxygen saturation during the apnea test portion. If a monitor is not available, the vitals can be provided to the learner before entering the simulation. The results of the apnea test can be communicated to the learner through the intercom, telephone, or written report.
- Gown.
- Q-tips.
- Tongue depressor.
- Pen light.
- Institutional checklist for determination of brain death.

Personnel
A member of the research team acted as the confederate (i.e., patient's spouse). Given that this role is a family member, they need not have an in-depth understanding of the nuances of brain death. In fact, since they portray a member of the lay community, it may improve the simulation if they have an incomplete understanding of brain death so that it makes the encounter more realistic. This role could effectively be portrayed with a standardized patient (Appendix B). A simulation technician was responsible for ensuring that the mannequin displayed clinical signs consistent with brain death and altering the respiratory rate and blood oxygen saturation during the apnea test. A clinical ethicist with expertise in neuroethics and a neurologist with expertise in brain death examinations observed the simulation from the control room through a one-way mirror.

Implementation
Implementation of the simulation followed the recommendations of Hocker and Wijdicks, which involved a prebrief-simulation-debrief-evaluation structure.[15]

Before simulation training, all participants received a didactic session (5-10 minutes) on the history of brain death, the dead donor rule, and the content of the Uniform Determination of Death Act (Appendix A). The trainees were presented with a simulated brain death case (Appendix C). Subsequently, one trainee volunteered to complete the case in the simulated ICU. The room was set up with a high-fidelity patient simulator (SimMan 3G Mannequin), standard ICU equipment (ventilator and monitor), and instruments needed to conduct a brain death exam (tongue depressor, pen light, gown, Q-tips, and institutional checklist for determination of brain death).

The volunteer trainee was informed that they should enter the room, discuss the brain death examination with the spouse and respond to any questions they have, then conduct a clinical examination for brain death, followed by a discussion of their findings with the patient's spouse. The trainee was provided with the WBUH institutional brain death policy that included the clinical criteria for determining brain death (Appendix D). Trainees should use their own institutional checklists during this portion of the simulation. A neurologist was always available during the debrief session in person or by phone to answer any questions about the clinical exam for brain death. Physical exam findings were not given to the trainee, as neither a brain-dead person nor a mannequin would react to physical examination (e.g., brain steam reflexes, withdrawal from painful stimuli, cold caloric test). The spouse was primed to challenge the trainee if medical jargon was used to deliver their findings. They expressed concerns and objections to the brain death examination and removal of medical support devices throughout the simulation, as per Appendix B. The trainee would be given some time to respond before the simulation ended. The simulation portion required 10-15 minutes to complete.

Debriefing
After completing the simulated case, the participating trainee, onlooking trainees, and confederate debriefed the simulation with two content experts—a clinical ethicist with expertise in neuroethics and a neurologist with expertise in brain death examinations (Appendix E). We highly recommend the presence of at least one facilitator who has had experience with performing a brain death examination and communicating its findings. We found that the anecdotal experience provided by the facilitators during the debrief sessions offered valuable guidance to the trainees. The debrief lasted the remaining time of the session (30 minutes). The 3D Model of Debriefing was used to guide the debriefing process and involved three steps: Defusing, Discovering, and Deepening.[16] Topics covered during the discovering step included:

1. How to perform a clinical examination for brain death.
2. Clinical ethics challenges and consensus positions.
3. Communication best practices.
4. Discussion of three competing philosophical views of death and the proposal for a conscience clause.

Assessment
We developed a 9-item survey focused on three domains: confidence in diagnosis and communication, understanding and appreciation for the philosophical basis of death, as well as attitudes toward nonstandard views of death (Appendix F). Given

Copyright © 2024 Ludka et al. This is an open-access publication distributed under the terms of the Creative Commons Attribution license.

that the simulation was developed for medical trainees who may not have been previously exposed to brain death, we wanted to understand how confident they were with performing the brain death examination and communicating a diagnosis of death. Furthermore, we opine that in order to facilitate a discussion of brain death effectively, one must have an understanding of the philosophical basis of brain death as well as nonstandard views of death. The survey was distributed before and after the simulation. All items were scored on a 5-point Likert scale (1 = *strongly disagree*, 5 = *strongly agree*. Two optional questions allowed trainees to give feedback to the facilitators. Shapiro-Wilk tests on the distribution of pre- and postsession responses for each item indicated significant skewness ($p < .05$ for each item). Therefore, a Wilcoxon signed-rank test was used to compare pre- and postintervention scores. The Mann-Whitney *U* test was used to compare scores between nominal variables. A Bonferroni correction was applied to account for multiple comparisons. Statistical significance was assessed at $\alpha = .05$.

Results

There were seven simulations with a total of 35 medical trainees that occurred between August 2022 to March 2023. All trainees who participated in the simulation took the survey. Group sizes ranged from three to seven with a median number of four trainees present for each session. The cohorts represented a wide range of both medical specialty and years of training: pulmonary and critical care fellowship, internal medicine residency, emergency medicine residency, anesthesiology residency, transitional year, internal medicine-pediatrics residency, and fourth-year medical students (Table 1). After the intervention, trainees were more confident in their ability to perform a brain death examination, talking to families about brain death, and responding to common ethical issues that arise in the context of brain death (Table 2). They also reported a greater understanding of the dead donor rule, the definition of death as described in the Uniform Determination of Death Act (UDDA), and nonstandard views of death (Table 2). While trainees reported a greater appreciation for the philosophical basis for disagreeing with brain death, they reported no changes in attitude with families who have either a circulatory or high brain view of death (Table 2). Both before and after the intervention, trainees were less frustrated with families with a high brain view of death than they were with families with a circulatory view. In the case of circulatory death, 23% (eight of 35) of trainees became more frustrated with families while 20% (seven of 35) became less frustrated following the simulation. In the case of high brain death, 14% (five of 35) of trainees became more frustrated with families and 14% (five of 35) became less frustrated. The

Table 1. Participant Demographics and Previous Experience With Brain Death ($N = 35$)

Demographics	No.	%
Gender		
Female	17	49
Male	17	49
Prefer not to answer	1	2
Program		
Internal medicine residency	19	54
Emergency medicine residency	6	17
Medical student	4	11
Pulmonary and critical care fellowship	3	9
Medicine-pediatrics residency	1	3
Anesthesiology residency	1	3
Transitional year	1	3
Stage of training		
Fourth year medical school	4	11
PGY 1	10	29
PGY 2	13	37
PGY 3	5	14
F1	2	6
F2	0	0
F3	1	3
Have previously performed brain death examination		
No	25	71
Yes	10	29

Abbreviations: PGY, postgraduate year; F, fellowship year.

mean number of years in training was higher among those who had previously performed a brain death examination ($\bar{x} = 2.4$) compared to those who had not ($\bar{x} = 1.6$); however, this was not statistically significant. Individuals who had previously performed a brain death examination were equivalent to those who had not previously performed a brain death examination on all items except for confidence in performing brain death examinations, where they were significantly more confident ($p < .001$). Overall, trainees were pleased with the session. The interactive nature of the training was cited by many trainees as being their favorite element.

We learned valuable lessons while we implemented this simulation. First, we learned that trainees are uncomfortable with surrogate decision-makers who do not consent to brain death examinations. In each simulation, the facilitator had to speak through the intercom to tell the trainee to proceed with the examination to keep the simulation on time. In the initial simulations, we had the spouse ask questions while the trainee performed the examination. We found that it was best to limit the spouse's comments during the examination (one to three comments) to allow time for the trainee to finish the examination and discuss the results. If the facilitator anticipated the simulation running short on time, they would communicate with the confederate via headset to ask that they keep their comments brief. While not mandatory, it was beneficial to have trainees or facilitators who have experience with brain death examinations

Copyright © 2024 Ludka et al. This is an open-access publication distributed under the terms of the Creative Commons Attribution license.

Table 2. Participant Survey Responses for Understanding, Attitudes, and Confidence, Pre- Versus Postsimulation Participation[a]

Survey Component	Survey Item	Preintervention *Mdn* (IQR)	Postintervention *Mdn* (IQR)	p^b
Understanding	High-brain and circulatory views of death	2 (1–4)	4 (4–5)	<.001
	Dead donor rule	2 (2–4)	4 (4–4)	<.001
	Criteria for death outlined in the UDDA	2 (1–4)	4 (4–5)	<.001
Attitudes	Appreciation for the philosophical basis of defining death	3 (3–4)	4 (4–4)	<.001
	Frustration towards families who hold a circulatory view of death	3 (2–4)	3 (2–4)	.72
	Frustration toward families who hold a high-brain view of death	2 (2–3)	2 (2–3)	.52
Confidence	Responding to common ethical issues that arise in the context of brain death	3 (2–4)	4 (3–4)	<.001
	Communicating with families about brain death	3 (2–4)	4 (3–4)	<.001
	Performing brain death examination	3 (2–3)	4 (3–4)	<.001

Abbreviation: UDDA, Uniform Determination of Death Act.
[a]Survey items were scored on a 5-point Likert scale (1 = *strongly disagree*, 5 = *strongly agree*).
[b]Statistics were calculated using a Wilcoxon signed rank test; *p* values include Bonferroni adjustments for multiple comparisons.

and end-of-life conversations. Sharing their experiences added valuable clinical context for the more theoretical concepts covered in the debrief (e.g., proper descriptive language of death, common objections to brain death).

Discussion

We successfully implemented a simulation on the history, ethics, clinical examination, communication challenges, and proposed conscience clause for brain death. Although trainees gained experience in the clinical examination for brain death, the focus of the didactic, simulation, and debrief was on the other aspects (e.g., historical, ethical, communication) of these cases. Placing a trainee in the simulation and having the others observe provided an opportunity for the other trainees to provide feedback on their proficiency regarding communicating with a family member in this situation. We observed that this awareness led to increased interest in discussion and receiving information on best practices in the debrief. Proposing various points in the debrief for discussion before providing best practices or consensus positions gave a chance for trainees to experience the complexity of these issues through lively discussion while also providing guidance for future practice. Moreover, the diverse composition of trainees that attended our sessions provided an opportunity for peer-to-peer teaching across both medical disciplines and levels of experience.

Using simulation to teach brain death, and its attendant ethical issues, is not a one-size-fits-all approach. Although our simulation was geared toward a diverse range of medical specialties, it could be tailored toward one specialty. For example, the case could be changed to include a neurosurgical intervention to focus on neurosurgery trainees or a complicated metabolic derangement to focus on internal medicine trainees. Furthermore, educational innovations aimed toward an increased understanding of brain death and the ethical issues that surround it should not just be for medical students, residents, and fellows. There are a multitude of professionals that work in the intensive care units: attending physicians, nurses, advanced practice providers, and social workers. Future simulations may be tailored toward the role each of these professionals have in brain death diagnosis and communication.

While a 1-hour session has some limitations, it has the distinct advantage of providing the salient information regarding brain death. The medical students and residents rotate through the intensive care unit on a monthly basis at WBUH. We found that a single, 1-hour session trainees could complete while in their ICU month would be most beneficial, as this is the part of their training where they are most likely to interact with patients with severe brain injuries. While a longitudinal curriculum may have provided more opportunities for trainees to participate in the actual simulation, it would not have aligned with their current clinical training, as they would no longer be rotating through the ICU. Future research into knowledge retention after attending one-time versus distributed educational innovations may be warranted to determine the optimal model for postgraduate education.

Our data indicate that despite no group change in frustration with families with nonstandard views, individuals shifted both toward greater and lesser frustration in both cases. It has been previously reported that educational innovations on the ethics of brain death can polarize second-year medical students into becoming more or less frustrated with those who espouse nonstandard views of death, and that students fall into one of two categories; death deontologists or death consequentialists.[17] Death deontologists ground their views of death in principles, (e.g., respect for persons or tolerance for nonstandard views), whereas consequentialists view death as it operates within the larger sociocultural context, (e.g., appealing to concepts like medical futility or distributive justice). Further research into the

Copyright © 2024 Ludka et al. This is an open-access publication distributed under the terms of the Creative Commons Attribution license.

impact that various forms of simulation have on the knowledge, skills, and attitudes of medical providers is needed to optimize interventions on the diagnosis, communication, and ethics of brain death.

This study has elements that limit its generalizability. WBUH is home to the Eugene and Marcia Applebaum Simulation Learning Institute (SLI) that comes equipped with high-fidelity mannequins, conference rooms with audio-visual streaming, one-way mirrors that allow for observation, and headsets that allow for communication between confederates and trainees. Such resources may not be available at other institutions. The facilitators also had significant legal, clinical, and ethical experience with brain death. Furthermore, although the trainees and clinical faculty told the facilitators that the training was helpful, this was only anecdotal. There was no follow-up with the trainees to investigate how well the information was retained. Future studies investigating the impact of simulation education would benefit from such long-term follow up. Also, the survey results are not parsed into who did and who did not participate in the simulation. Investigating the impact of observing a simulation versus participating in a simulation may be warranted given the time constraints inherent in having multiple trainees participate in simulations. Also of note, the survey used was developed by our research team and did not represent a validated tool. Development of a validated tool would be a valuable addition to the future study of brain death simulations. Lastly, although the three views of death outline in this simulation are the most common, they are not an exhaustive list on the views of death. A definition of death requires both scientific and philosophical claims, and as such, there is a diverse set of cultural perspectives on death.[18] Further simulation training that incorporates views outside of the whole-brain, high-brain, and circulatory view is warranted to help trainees prepare for work in a pluralistic society.

Appendices

A. Prebrief Instructions and Presentation.pptx

B. Standardized Patient Case Development Tool.docx

C. Simulation Case.docx

D. WBUH Checklist for Determining Brain Death.docx

E. Instructions for Debrief.docx

F. Questionnaire.docx

All appendices are peer reviewed as integral parts of the Original Publication.

Nicholas Ludka: Fourth-Year Medical Student, Oakland University William Beaumont School of Medicine; ORCID: https://orcid.org/0000-0003-0410-7617

Ngan Nguyen, PhD: Assistant Professor, Department of Foundational Medical Studies, Oakland University William Beaumont School of Medicine

Daniel Menkes, MD, MHSA: Professor, Department of Neurology, Oakland University William Beaumont School of Medicine

Abram Brummett, PhD, HEC-C: Assistant Professor, Department of Foundational Medical Studies, Oakland University William Beaumont School of Medicine

Disclosures
None to report.

Funding/Support
None to report.

Ethical Approval
Reported as not applicable.

References

1. Greer DM, Shemie SD, Lewis A, et al. Determination of brain death/death by neurologic criteria: the World Brain Death Project. *JAMA*. 2020;324(11):1078. https://doi.org/10.1001/jama.2020.11586
2. Kramer NM, O'Mahony S, Deamant C. Brain death determination and communication: an innovative approach using simulation and standardized patients. *J Pain Symptom Manage*. 2022;63(6): e765-e772. https://doi.org/10.1016/j.jpainsymman.2022.01.020
3. Chen PM, Trando A, LaBuzetta JN. Simulation-based training improves fellows' competence in brain death discussion and declaration. *Neurologist*. 2022;27(1):6-10. https://doi.org/10.1097/NRL.0000000000000354
4. Afif IN, Goldberg AJ, Zhao H, et al. Formal training improves resident understanding and communication regarding brain death/death by neurologic criteria. *J Surg Educ*. 2022;79(1):198-205. https://doi.org/10.1016/j.jsurg.2021.08.018
5. Fourcand F, Barratt DM. How educators can help prevent false brain death diagnoses. *AMA J Ethics*. 2020;22(12):E1010-1018. https://doi.org/10.1001/amajethics.2020.1010
6. Barnes E, Greer D. Inconsistency in brain death determination should not be tolerated. *AMA J Ethics*. 2020;22(12): E1027-1032. https://doi.org/10.1001/amajethics.2020.1027
7. Russell JA, Epstein LG, Greer DM, Kirschen M, Rubin MA, Lewis A. Brain death, the determination of brain death, and member guidance for brain death accommodation requests: AAN position statement. *Neurology*. 2019;92(5):228-232. https://doi.org/10.1212/WNL.0000000000006750

Copyright © 2024 Ludka et al. This is an open-access publication distributed under the terms of the Creative Commons Attribution license.

8. Wijdicks EFM, Varelas PN, Gronseth GS, Greer DM. Evidence-based guideline update: determining brain death in adults: report of the Quality Standards Subcommittee of the American Academy of Neurology. *Neurology*. 2010;74(23): 1911-1918. https://doi.org/10.1212/WNL.0b013e3181e242a8

9. Greer DM, Wang HH, Robinson JD, Varelas PN, Henderson GV, Wijdicks EFM. Variability of brain death policies in the United States. *JAMA Neurol*. 2016;73(2):213-218. https://doi.org/10.1001/jamaneurol.2015.3943

10. MacDougall BJ, Robinson JD, Kappus L, Sudikoff SN, Greer DM. Simulation-based training in brain death determination. *Neurocrit Care*. 2014;21(3):383-391. https://doi.org/10.1007/s12028-014-9975-x

11. Douglas P, Goldschmidt C, McCoyd M, Schneck M. Simulation-based training in brain death determination incorporating family discussion. *J Grad Med Educ*. 2018; 10(5):553-558. https://doi.org/10.4300/JGME-D-18-00185.1

12. Morris NA, Zimmerman EE, Pozner CN, Henderson GV, Milligan TA. Brain death determination: an interprofessional simulation to determine brain death and communicate with families focused on neurology residents. *MedEdPORTAL*. 2020;16:10978. https://doi.org/10.15766/mep_2374-8265.10978

13. Barratt D. Coma simulation enhances learning of brainstem reflexes. *MedEdPORTAL*. 2014;10:9669. https://doi.org/10.15766/mep_2374-8265.9669

14. Morrison WE, Kirschen MP. A taxonomy of objections to brain death determination. *Neurocrit Care*. 2022;37(2):369-371. https://doi.org/10.1007/s12028-022-01580-6

15. Hocker S, Wijdicks EFM. Simulation training in brain death determination. *Semin Neurol*. 2015;35(2):180-188. https://doi.org/10.1055/s-0035-1547535

16. Zigmont JJ, Kappus LJ, Sudikoff SN. The 3D model of debriefing: defusing, discovering, and deepening. *Semin Perinatol*. 2011;35(2):52-58. https://doi.org/10.1053/j.semperi.2011.01.003

17. Ludka N, Brummett A, Wasserman JA. Education research: changes in medical students' knowledge and attitudes toward clinical death after teaching the philosophy of death. *Neurol Educ*. 2023;2(2):e200055. https://doi.org/10.1212/NE9.0000000000200055

18. Veatch RM, Ross LF. *Defining Death: The Case for Choice*. Georgetown University Press; 2016.

Received: June 29, 2023
Accepted: May 9, 2024
Published: September 26, 2024

Copyright © 2024 Ludka et al. This is an open-access publication distributed under the terms of the Creative Commons Attribution license.
